# Current and Perspective Sensing Methods for Monkeypox Virus

**DOI:** 10.3390/bioengineering9100571

**Published:** 2022-10-18

**Authors:** Ijaz Gul, Changyue Liu, Xi Yuan, Zhicheng Du, Shiyao Zhai, Zhengyang Lei, Qun Chen, Muhammad Akmal Raheem, Qian He, Qiuyue Hu, Chufan Xiao, Zhang Haihui, Runming Wang, Sanyang Han, Ke Du, Dongmei Yu, Can Yang Zhang, Peiwu Qin

**Affiliations:** 1 Institute of Biopharmaceutical and Health Engineering, Tsinghua Shenzhen International Graduate School, Tsinghua University, Shenzhen 518055, China; gul.ijaz@sz.tsinghua.edu.cn (I.G.); liu-cy20@mails.tsinghua.edu.cn (C.L.); yuanx20@mails.tsinghua.edu.cn (X.Y.); duzc21@mails.tsinghua.edu.cn (Z.D.); dsy20@mails.tsinghua.edu.cn (S.Z.); leizy21@mails.tsinghua.edu.cn (Z.L.); chen-q17@mails.tsinghua.edu.cn (Q.C.); dr.akmalraheem1362@stu.ahau.edu.cn (M.A.R.); he.qian@sz.tsinghua.edu.cn (Q.H.); qy-hu20@mails.tsingua.edu.cn (Q.H.); xiaocf21@mails.tsinghua.edu.cn (C.X.); zhanghaihui@sz.tsinghua.edu.cn (Z.H.); runmingwang@sz.tsinghua.edu.cn (R.W.); hansanyang@sz.tsinghua.edu.cn (S.H.); 2 Tsinghua-Berkeley Shenzhen Institute, Tsinghua Shenzhen International Graduate School, Tsinghua University, Shenzhen 518055, China; 3 Chemical and Environmental Engineering, University of California, Riverside, CA 92521, USA; ke.du@rit.edu; 4 School of Mechanical, Electrical & Information Engineering, Shandong University, Weihai 264209, China

**Keywords:** monkeypox, real-time PCR, LAMP, RPA, immunoassay, diagnosis

## Abstract

The outbreak of the monkeypox virus (MPXV) in non-endemic countries is an emerging global health threat and may have an economic impact if proactive actions are not taken. As shown by the COVID-19 pandemic, rapid, accurate, and cost-effective virus detection techniques play a pivotal role in disease diagnosis and control. Considering the sudden multicountry MPXV outbreak, a critical evaluation of the MPXV detection approaches would be a timely addition to the endeavors in progress for MPXV control and prevention. Herein, we evaluate the current MPXV detection methods, discuss their pros and cons, and provide recommended solutions to the problems. We review the traditional and emerging nucleic acid detection approaches, immunodiagnostics, whole-particle detection, and imaging-based MPXV detection techniques. The insights provided in this article will help researchers to develop novel techniques for the diagnosis of MPXV.

## 1. Introduction

The ongoing COVID-19 pandemic and the recent monkeypox virus (MPXV) outbreak reflect the need for a viable global healthcare system. Almost every country is now globally connected, and infectious disease outbreaks have become a constant global threat, necessitating proactive measures [1]. MPXV is an adenovirus with a double-stranded DNA genome, belonging to the family *Poxviridae*, subfamily *Chordopoxvirinae*, and the genus *Orthopoxvirus* [2,3]. MPXV was first reported in 1958 after two pox-like disease outbreaks occurred in monkeys [4]. The original source of MPXV is unknown. Rodents likely harbor the virus [5], leading to spillover events. The case of human infection by MPXV was first reported in humans in in the Democratic Republic of the Congo in 1970 [6]. The transmission of MPXV from animals to humans may occur by direct or indirect contact with infected organisms (live or dead), while close contact with symptomatic cases is thought to be the main human-to-human transmission mode [5]. MPXV infection in asymptomatic or undiagnosed (where signs and symptoms overlap with other diseases) men who have sex with men (MSM) was also reported in a recent case study [5]. In recent MPXV outbreaks, the MPXV cases were predominantly reported in homosexual or bisexual males [7]. The approximate incubation period of MPXV is about 5–21 days [8,9]. However, an incubation period of 3–20 days was also reported [10].

Recently, a multicountry monkeypox outbreak was reported to the World Health Organization (WHO) by several non-endemic countries. Since January 2022, and as of 14 September 2022, about 103 member states from six regions have reported a total of 59,147 confirmed cases of MPXV and 22 deaths [7]. MPXV has been declared a global health emergency by the WHO [11].

Diagnostic methods play a pivotal role in infectious disease control and monitoring. Nucleic acid amplification assays (NAAa), sequencing, and serological tests have been developed for MPXV (Table 1). Quantitative polymerase chain reaction (qPCR) and sequencing are common MPXV diagnostics [12]. In addition to PCR and sequencing, isothermal amplification methods have been developed in an effort to complement the PCR-based approaches [13]. However, their clinical implementation has not yet been demonstrated. Though isothermal amplification methods do not rely on thermal cyclers and reduce diagnostic costs, these methods have certain limitations in terms of selectivity and operational ease [13], providing room for future developments. Since MPXV has spread to many demographics, a review of MPXV detection techniques and possible development opportunities could be a timely addition to the fight against MPXV.

Herein, we highlight the MPXV detection modalities and discuss challenges and opportunities. We start with a brief introduction to MPXV, including its genome organization, followed by a detailed discussion of monkeypox diagnostic approaches. The limitations and possible solutions are delineated.

## 2. Overview of Monkeypox Virus

MPXV, together with other orthopoxviruses, is a complex virus and has one of the largest viral genomes [14]. Under an electron microscope, MPXV and other poxviruses show a brick-shaped geometry [15,16]. The size range of the monkeypox virus is 200 to 250 nm [17]. The genome size of MPXV is about 197 kbp [18]. The virion genome contains inverted tandem repeats, tandem repeats, open reading frames, and hairpin loops [19]. The MPXV genome has a conserved central genomic region harboring housekeeping genes, while variable regions on both termini are involved in virus pathogenesis [15,19,20,21].

MPXV is genetically divided into two main clades: clade 1, formerly known as the Congo Basin or Central African (CA) clade, and clade 2, formerly designated as the West African (WA) clade [21]. The fatality rate of the WA clade is relatively lower. Conversely, the CA clade is more virulent (the fatality rate is about 11%) and is potentially more transmissible [22]. Recently, the WHO convened global experts on the nomenclature of virus variants or clades [23]. A consensus was reached. According to the consensus, the MPXV genome is divided into two clades, viz. clade I and clade II. Clade II is divided into subclades: clade IIa and clade IIb (the currently circulating clade) [23]. Clade I corresponds to the genome from the CA clade, while clade II corresponds to the WA clade [24].

**Figure 1:**
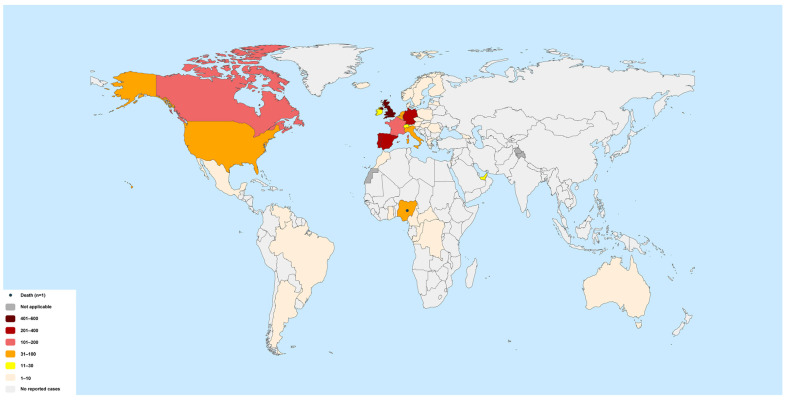
Multicountry MPXV outbreak. The figure shows the countries where the recent outbreak was initially reported. The numbers indicate the total number of cases in each country during January 2022–June 2022. Redrawn from Ref. [25].

Initially, most of the cases were concentrated in the European region (Figure 1) [25], but the virus is now increasingly spreading to other non-endemic countries. A total of 28 deaths have been reported so far [26]. Among the globally infected countries, countries from the American and European regions are the most affected [26].

## 3. Monkeypox Diagnosis Approaches

**Figure 2:**
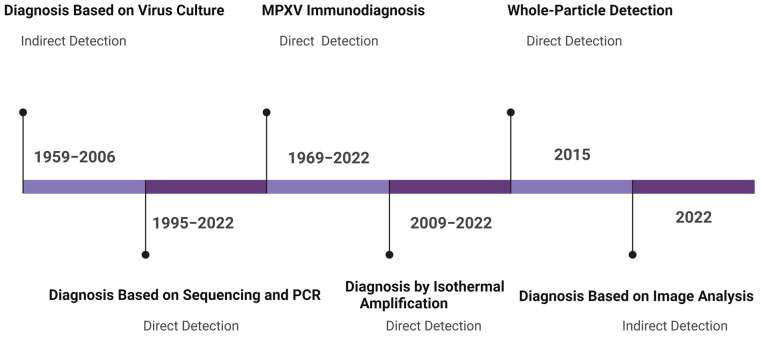
Overview of MPXV diagnostics. The years indicate the time frames of the published articles discussed in this manuscript.

Since MPXV is a re-emerging virus, a number of MPXV detection modalities have been developed since its discovery (Figure 2).

### 3.1. Indirect Detection

Indirect detection is based on virus-induced morphological changes to host cells or membranes. 

#### 3.1.1. Monkeypox Diagnosis Based on Virus Culture

Some viruses can induce macroscopic lesions (called pocks) on the chick chorioallantoic membrane (CAM). The pattern of pock formation, the time required for pock formation, and the size of the pock have been explored to differentiate different poxvirus infections, including MPXV [27,28]. The morphological changes can be observed with a microscope or the naked eye. For instance, when CAM was inoculated with MPXV, the pocks were visible and could be reckoned with the naked eye [28]. However, detection solely based on the above-mentioned characteristics may not be sufficient for an accurate diagnosis due to overlapping signs and symptoms with other diseases.

Monkeypox isolates are grown in RK13 cells [29], where cytopathic effects are observed within 24–48 h of infection. The major drawback of culture-based virus diagnosis is the prolonged assay time [30], which is not suitable for mass testing scenarios. Further, virus culture methods need biosafety level 3 (BSL3) labs and pose a risk of laboratory-acquired infections [31]. Shell vial culture (SVC) has been developed as an alternative culture method for the rapid in vitro detection of MPXV and other viruses [30]. In this method, a cell monolayer is grown on a cover slip in a shell vial culture tube, and the specimen is inoculated on the monolayer, followed by low-speed centrifugation and immunofluorescence-based detection. The low-speed centrifugation step is introduced to enhance the virus’s infectivity. The mechanical force resulting from low-speed centrifugation is thought to cause cell trauma, which subsequently enhances viral entry into cells, resulting in a reduced cell infection time [32]. 

#### 3.1.2. Diagnosis Based on Image Analysis

**Figure 3:**
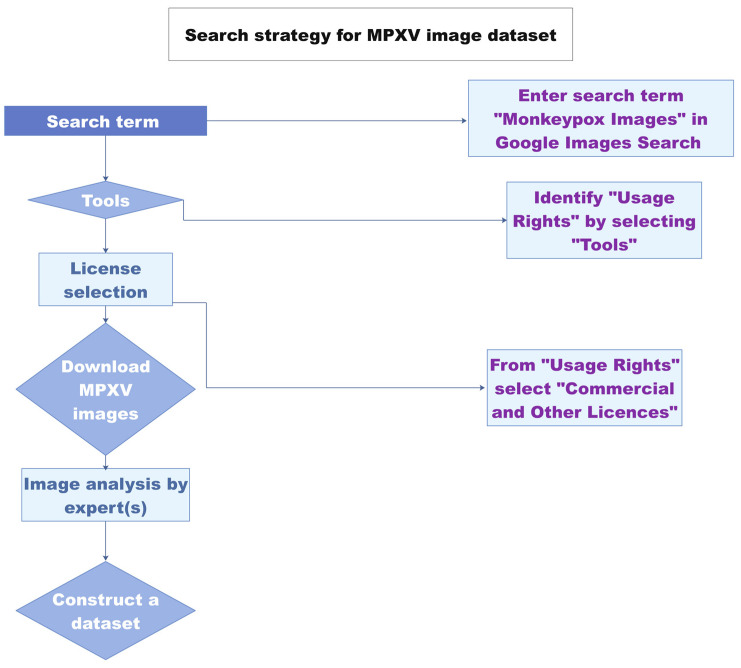
Schematic of the image-based MPXV detection workflow. Redrawn from Ref. [35] with permission from the author. The source content is licensed under a Creative Commons Attributions 4.0 international license.

Image digitalization has already gained momentum for infectious disease diagnosis and monitoring. Chatbots have been developed for disease diagnostic evaluation and the recommendation of immediate measures in case a patient contracts SARS-CoV-2 [33]. A monkeypox image dataset was constructed comprising 43 original images and 587 images obtained after data augmentation [34] (Figure 3). Using the newly developed “Monkeypox 2022” dataset, an image classification model was proposed [35]. The study paves the way towards the development of image-analysis-based tools for monkeypox virus detection. The images used in the dataset are from previous outbreaks. The classic MPXV cases were characterized by a generalized rash. In contrast, most of the cases in the current outbreak have localized lesions in anogenital and genitourinary areas [36,37]. Since many recent MPXV-infected cutaneous images have been reported, the updated dataset may have added value to the above MPXV 2022 image dataset.

### 3.2. Direct Detection

In the case of direct detection, nucleic acid and protein components of the virus are detected without the need for a pathogen culture. Molecular detection, immunodiagnostics, and sequencing are widely explored direct detection approaches.

#### 3.2.1. Monkeypox Immunodiagnostics

The hemagglutination test is a simple and cost-effective approach for virus detection. The test is based on the agglutination of erythrocytes in the presence of a virus [30]. The hemagglutination mechanism led to the development of another assay called the hemagglutination inhibition (HI) assay [38]. The HI approach relies on virus-specific antibodies to detect viral antigens. The MPXV strains are tested using hemagglutination and HI tests [28]. The test cannot differentiate MPXV from the variola and vaccinia viruses but can differentiate cowpox from MPXV and can be used to estimate the evolutionary relationships of viral strains or species.

The enzyme-linked immunosorbent assay is a widely used protein detection method [39]. A commercially available Orthopox BioThreat^®^ Alert Assay for orthopox virus (OPV) detection is a reliable OPV detection method [40]. This antibody-based lateral flow assay captures virus antigens and detects the viral load at 10^4^ PFU/mL. The surface protein A27 was found to be the most immunogenic protein for virus particle capture and detection [41]. After a comprehensive screening of A27-binding antibodies, an ELISA approach was developed for orthopoxviruses, including MPXV. The method’s detection limit is 1× 10^3^ PFU/mL. In a similar line of work, an ABICAP (Antibody Immuno Column for Analytical Processes) immunofiltration system was developed by Stern et al. The system has an OPV detection sensitivity of 10^4^ PFU/mL with an assay time of 45 min [42]. A dot immunoassay based on protein array technology can detect MPXV in a concentration range of 10^3^–10^4^ PFU/mL within 39 min [43]. Recently, Ulaeto et al. described the characteristics of an LFA for the detection of orthopoxviruses [44]. The assay detects vaccinia virus samples spiked in human saliva and clinical sample buffer with a detection limit of between 10^4^ and 10^5^ PFU/mL within 20 min. Since this assay detects orthopoxviruses, the test can be further explored for MPXV detection in real samples. Combining the clinical presentation of MPXV with the LFA test could provide a rapid MPXV detection tool. All of the above-mentioned immunodetection modalities are suitable for generic orthopox virus detection applications, but none of them are specific for MPXV.

#### 3.2.2. Whole-Particle Detection

Finding biomarkers for a newly emerged virus is challenging and may hamper the direct implementation of routine diagnostic methods. In this regard, whole-particle detection using electron microscopy (EM) is a powerful alternative [45]. Transmission electron microscopy is a good first step for the detection of viruses, as it provides information about the shape and amount of viral load with a small sample volume [46]. The use of virus-specific antibodies in immunoelectron microscopy (IEM) further improves the detection accuracy of EM [47]. EM has been used to detect monkeypox and other orthopoxviruses [48]. Although EM is suitable for the laboratory validation of the virus detection results, the approach has certain limitations, such as the high cost of the instrument, the requirement of highly trained staff, and low sample throughput [48].

#### 3.2.3. Detection by Genome Sequencing

Genome sequencing is the gold standard to identify novel or mutated viruses. Genome sequencing not only identifies the target virus but may pinpoint the presence of other viruses in the sample that can help to create a treatment plan for a particular disease. MPXV detection based on qPCR coupled with genome sequencing has been reported [49]. To date, 200 genome sequences of MPXV isolates from recent outbreaks in non-endemic countries have been reported [50]. Whole-genome sequencing is a time-consuming process and requires expensive instruments, trained staff, and skilled bioinformaticians for computational analyses. These limitations need to be overcome to harness the potential of genome sequencing approaches.

#### 3.2.4. Monkeypox Virus Detection Based on PCR

The polymerase chain reaction (PCR) is widely regarded as the gold standard for nucleic acid detection. According to WHO recommendations, PCR (conventional or real-time) is a standard method for MPXV laboratory validation [51]. The detection can be combined with sequencing or other orthopox detection assays [19]. Conventional PCR-based MPXV detection involves PCR amplification and restriction digestion of the PCR-amplified fragments to identify MPXV based on restriction fragment length polymorphisms.

A hemagglutinin PCR (HA-PCR) assay was developed based on MPXV-specific primers coupled with *Taq*I restriction digestion [52]. The method could not distinguish different MPXV isolates. To improve the detection accuracy of the PCR assay, an A-type inclusion body protein (ATI) gene has been used to detect MPXV and other orthopoxviruses based on PCR-based gene amplification and *Xba*I digestion [53,54]. The method can differentiate MPXV strains based on restriction digestion. In another development, the open reading frame (ORF) of the ATI gene was identified, sequenced, and compared with other related poxviruses [55]. Unique deletions were found in the OFR of MPXV and were harnessed for the specific detection of the MPXV ATI gene. This PCR method differentiates 19 MPXV strains. The specificity was confirmed by *Bgl*II restriction digestion.

Compared to traditional PCR, real-time PCR is rapid and sensitive. Due to the low GC content and almost 90% genome identity with other Eurasian *orthopoxviruses*, designing an MPXV-specific TaqMan assay is challenging. Li et al. developed a real-time PCR assay where minor-groove-binding protein-based (MGB) probes were developed [56]. The use of MGB stabilizes probe–template interactions, enables the use of small probe sequences for single-nucleotide polymorphism (SNP) detection, and enhances assay sensitivity and specificity [57]. The method could detect 15 MPXV isolates at a 10 ng concentration. The assay efficiency with freshly diluted DNA is 97%, while it is reduced to 67% after multiple freeze–thaw cycles. These observations indicate that a fresh sample should be used in order to achieve maximum assay efficiency. The detection of MPXV and other orthopoxviruses based on melting-curve analysis (MCA) has also been reported [58,59,60]. Both clades (West African and Congo Basin) of MPXV have 99% sequence identity but are significantly different in terms of virulence [61]. It is a big challenge to develop a clade-specific real-time PCR detection approach due to the limited availability of unique sequences. In an effort to differentiate between isolates from the two different clades, the terminal genomic sequences of MPXV strains were analyzed [62]. Since the terminal sequences show relatively more sequence variability than the central genomic region and the G2R protein gene lies in the terminal genomic region, the G2R protein gene was chosen to design primers and probes for the West African MPXV specific assay called G2R-WA. No unique sequences were found in the G2R protein gene of the Congo Basin clade. Therefore, another gene, the C3L protein gene, is targeted for Congo Basin MPXV [62].

Multiplex detection can significantly reduce the misidentification of coexisting pathogens [63,64]. A multicolor, multiplex approach for MPXV detection was reported where MPXV was specifically detected in the presence of the variola virus (VARV) and the varicella-zoster virus (VZV) [65]. The target genes harboring unique sequences for MPXV, VARV, and VZV are F3L, B12R, and ORF38, respectively. The specificity of the developed approach is 100%, and LODs of 20 copies per reaction for MPXV and VARV and 50 copies per reaction for VZV were reported. The robustness of the approach was demonstrated by successfully detecting the different combinations of MPXV, VARV, and VZV samples.

The standard poxvirus detection approach combines the disease’s clinical symptoms with a generic poxvirus PCR assay, followed by a poxvirus-specific PCR assay [60]. These pan-pox real-time PCR methods are instrumental in the accurate diagnosis of poxvirus infection. Based on the GC content, the chordopoxviruses (poxviruses that infect vertebrates) of the subfamily *Chordopoxvirinae* have two distinct genome types: one genome type contains high GC content (>60%), while the other genome type is comprised of low GC content (30–40%) [66]. GC-content-based pan-pox PCR assays have been developed [66]. The assays are termed high-GC PCR and low-GC PCR assays. The developed PCR assays detected DNA samples from more than 150 isolates and strains of chordopoxviruses. The detection approach is based on conventional PCR, and PCR amplicons are evaluated by *Taq*I RFLP patterns. In a similar line of work, a real-time PCR assay for the universal detection of orthopoxviruses was reported [64]. The system was reported to be able to detect poxviruses excluded in a previous study [66] as well as those from the subfamily *Entomopoxvirinae*. This assay targets a 100 bp highly conserved sequence in the D6R gene of poxviruses. The specificity of the assay for vertebrate samples is 99.8%, while it is 99.7% for arthropod samples. The system is 100% sensitive for vertebrate samples and 86.6% sensitive for arthropod samples. The detection limits are reported to be 100 or 1000 copies per reaction, depending on the poxvirus species.

#### 3.2.5. Detection Based on Isothermal Amplification

**Figure 4:**
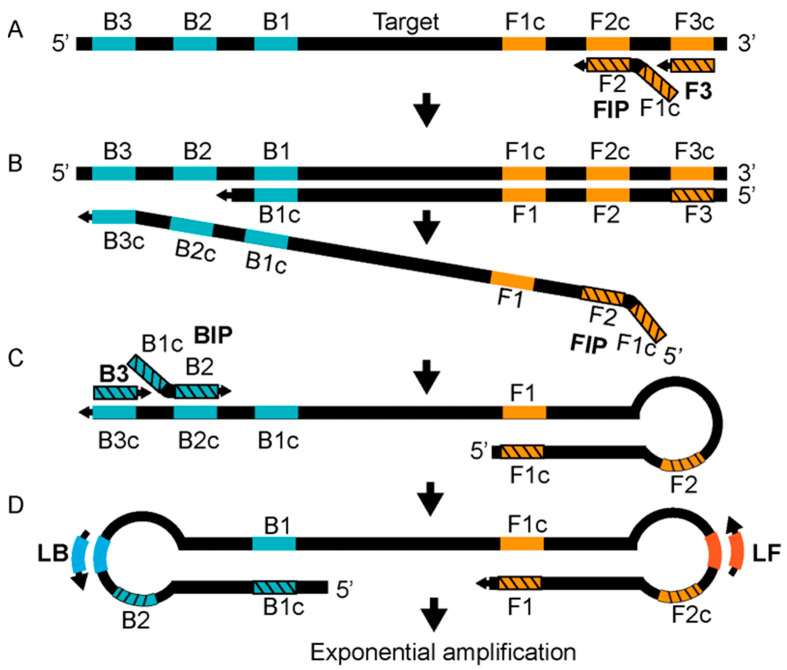
Reaction mechanism of LAMP. See text for details. Redrawn from Becherer et al., 2020, Ref. [67] © The Royal Society of Chemistry 2020, licensed under a Creative Commons Attributions-Noncommercial 3.0 unported license https://creativecommons.org/licenses/by-nc/3.0/. Accessed on 12 October 2022.

More than ten types of different isothermal amplification methods have been reported and demonstrated for nucleic detection [13]. Loop-mediated isothermal amplification (LAMP) and recombinase polymerase amplification (RPA) are well-explored isothermal nucleic acid amplification based virus detection methods [67]. The LAMP technology relies on two internal primers called the forward internal primer (FIP) and the backward internal primer (BIP), two outer primers known as the forward outer primer (F3) and the backward outer primer (B3), and a DNA polymerase with strand displacement activity [67]. The reaction is carried out at 60–65 °C. The amplification reaction is accelerated by using two loop primers, the forward loop (LF) and the backward loop primer (LB) [68]. The annealing of the FIP, which has two target sequences (separated by a spacer) complementary to the two different regions of the template, initiates strand synthesis and elongation (Figure 4A). Subsequently, the F3 primer displaces the FIP strand, producing a single-stranded DNA (ssDNA) strand that is used as a template by the BIP (Figure 4B). The BIP, which also has two target sequences complementary to the template DNA at two different regions, starts the strand elongation of the ssDNA template, which is later displaced by the B3 (Figure 4C). The 5′ and 3′ ends of the template DNA have inward complementary sequences, forming a stem-looped DNA that is exponentially amplified by loop primers (Figure 4C,D). LAMP-based MPXV-clade-specific assays have been developed where West African (the assay named W-LAMP) and Congo Basin MPXV (the assay named C-LAMP) clades are selectively detected [69]. A turbidimeter is used to analyze the LAMP reaction, and restriction digestion is used to confirm the LAMP products. A LAMP-based method for rapid MPXV detection was recently posted on a preprint server [70]. The assay was developed to detect MPXV clades. The method shows satisfactory sensitivity and response times.

**Figure 5:**
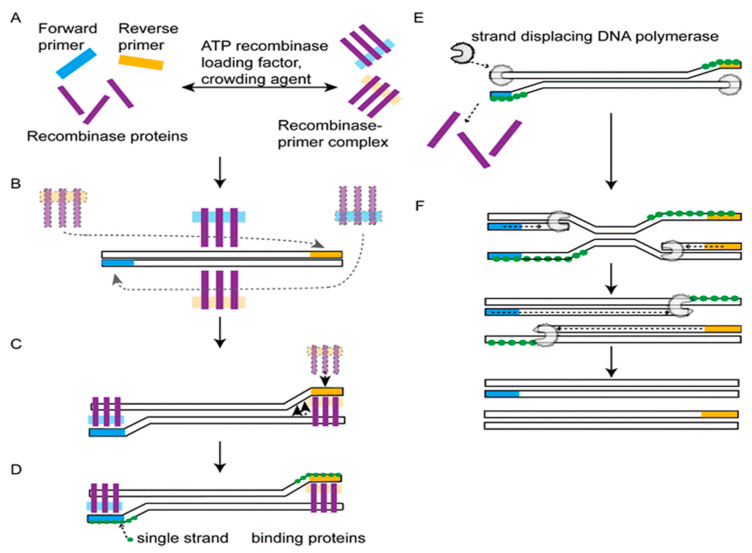
Reaction mechanism of RPA. Recombinase complexation with primer (**A**). Scanning of homologous sequences by recombinase–primer complex (**B**). Strand displacement by recombinase and primer insertion (**C**) and binding of single-strand-binding proteins to stabilize the primer binding (**D**). Recombinase disassembly and binding of strand-displacing DNA polymerase (**E**). Elongation reaction (**F**). Adapted from Ref. [71] with permission from Elsevier. Copyright © 2017 Elsevier B.V.

Although promising, the LAMP needs a 60-minute reaction time and six primers. Furthermore, primer design is relatively complex. To overcome these limitations, RPA has been proposed as an attractive alternative [71] (Figure 5). The RPA signal is detected by gel electrophoresis, real-time monitoring [72], or lateral flow assay [72]. In the case of real-time detection, the fluorogenic probe, along with the primers, is added to the reaction system where cleavage of the probe by exonuclease leads to a fluorescent signal. RPA-based MPXV detection shows satisfactory results with reduced assay times and reagent costs [73].

**Table 1:** Summary of MPXV diagnostic methods.

Sr. No	Assay Name	Target Gene	Primers’ Sequences	Probes’ Sequences	Detection Limit	Real-Sample Analysis	References
**1**	HA-PCR	HA gene	Forward: 5′-CTGATAATGTAGAAG AC -3′ Reverse: 5′-TTGTATTTACGTGGGTG-3′	NA	Not reported	Yes	[52]
**2**	ATI-PCR	ATI-gene	Forward: 5′-AATACAAGGAGGATCT-3′ Reverse: 5′-CTTAACTTTTTCTTTTTCTTTCTC-3′	NA	Not reported	Yes	[53]
**3**	MPXV PCR assay	ATI-gene	Forward: 5′-GAGAGAATCTCTTGATAT-3′ Reverse: 5′-ATTCTAGATTGTAATC-3′	NA	Not reported	Yes	[55]
**4**	Real-time PCR	B6R	Forward: 5′-ATTGGTCATTATTTTTGTCACAGGAACA-3′ Reverse: 5′-AATGGCGTTGACAATTATGGGTG-3′	5′-MGB/DarkQuencher-AGAGATTAGAAATA-3′-FAM	∼10 viral copies (2 fg)	Yes	[56]
**5**	Real-time PCR	G2R	Forward: 5′-CACACCGTCTCTTCCACAGA -3′ Reverse: 5′-GATACAGGTTAATTTCCACATCG -3′	5′-FAM AACCCGTCGTAACCAGCAATACATTT-3′-BHQ1	∼8.2 genome copies (1.7 fg)	Yes	[62]
**6**	Real-time PCR	G2R	Forward: 5′-TGTCTACCTGGATACAGAAAGCAA-3′ Reverse: 5′-GGCATCTCCGTTTAATACATTGAT -3′	5′-FAM-CCCATATATGCTAAATGTACCGGTACCGGA-3′-BHQ1	∼40.4 copies (9.46 fg)	Yes	[62]
**7**	Real-time PCR	F3L	Forward: 5′-CTCATTGATTTTTCG CGGGAT A-3′ Reverse: 5′-GACGATACTCCTCCT CGTTGGT-3′	5′-6FAM-CATCAGAATCTGTAGGCCGT-MGBNFQ-3′	11–55 fg (50–250 copies)	Yes	[74]
**8**	Real-time PCR	N3R	Forward: 5′-AACAACCGT CCTACA ATTAAA CAACA-3′ Reverse: 5′-CGCTATCGAACCATT TTTGTAGTCT-3′	5′-6FAM-TAT AAC GGC GAA GAA TAT ACT-MGBNFQ-3′	11–55 fg (50–250 copies)	Rodents	[74]
**9**	Real-time PCR	B7R	Forward: 5′-ACGTGTTAAACAATGGGTGATG-3′ Reverse: 5′-AACATTTCCATGAATCGTAGTCC-3′	5′-TAMRA-TGAATGAATGCGATACTGTATGTGTGGG-3′-BHQ2	50 copies per reaction	Yes	[75]
**10**	C-LAMP	D14L	FIP-C: 5′-TGGGAGCATTGTAACTTATAGTTGCCCTCCTGAACACATGACA-3′ F3-C: 5′-TGGGTGGATTGGACCATT-3′ BIP-C: 5′-ATCCTCGTATCCGTTATGTCTTCCCACCTATTTGCGAATCTGTT-3′ B3-C: 5′-ATGGTATGGAATCCTGAGG-3′ LOOP-F-C: 5′-GATATTCGTTGATTGGTAACTCTGG-3′ LOOP-C-C: 5′-GTTGGATATAGATGGAGGTGATTGG-3′	N/A	10^2.4^ copies per reaction	Yes	[69]
**11**	C-LAMP	ATI	FIP-W: 5′-CCGTTACCGTTTTTACAATCGTTAATCAATGCTGATATGGAAAAGAGA-3′ F3-W: 5′-TACAGTTGAACGACTGCG-3′ BIP-W: 5′-ATAGGCTAAAGACTAGAATCAGGGATTCTGATTCATCCTTTGAGAAG-3′ B3-W: 5′-AGTTCAGTTTTATATGCCGAAT-3′ LOOP-F-W: 5′-GATGTCTATCAAGATCCATGATTCT-3′ LOOP-C-W: 5′-TCTTGAACGATCGCTAGAGA-3′	N/A	10^3^ copies per reaction	Yes	[69]
**12**	RPA	G2R	Forward: 5′-AATAAACGGAAGAGATATAGCACCACATGCAC-3′ Reverse: 5′-GTGAGATGTAAAGGTATCCGAACCACACG-3′	5′-ACAGAAGCCGTAATCTATGTTGTCTATCGQTFCCTCCGGGAACTTA-3′	16 DNA molecules/μL	Yes	[73]

## 4. Wastewater-Based Epidemiology of MPXV

Wastewater epidemiology (WWE) is a relatively new approach and has the potential to achieve many ambitious objectives, such as determining the exposure of a particular community to an illicit drug, persistent pollutant, or any other hazardous material [76]. Wastewater fingerprinting might be a valuable tool to determine the viral load in a particular population in an epidemic outbreak where people share a sewage system, and water could be collected from a common sewage sampling point [77]. The unabated SARS-CoV-2 pandemic and the current multicountry MPXV outbreak indicate that the global healthcare system needs innovative disease monitoring tools such as smart diagnostics based on artificial intelligence, Internet of Things (IoT), machine learning, big data, and other related approaches. MPXV has been detected in various body fluids, such as urine, semen, saliva, nasopharynx fluid, serum, plasma, feces, and vaginal fluid [3,78]. The virus from infected individuals may be released into the environmental waters from skin flakes; by showering, urinating, or defecating; or from the release of seminal fluid in the water. Based on this assumption, Eline et al. recently assayed wastewater samples for MPXV detection in the Netherlands using PCR. The authors detected MPXV in many samples. How MPXV enters into the water is unknown. Further, animal reservoirs of MPXV may also contribute virus to the environmental waters. Further studies are needed to ascertain that the detected MPXV DNA is really from a human source. Another report also described MPXV DNA detection in environmental water samples [79]. In both reports, MPXV DNA in the solid fraction of wastewaters was reported to be higher than in the liquid fractions and could be used as a sample for virus detection. Since wastewater is a complex matrix, developing a standard method for wastewater-based virus detection seems challenging. Whether MPXV is persistent and infective in the water bodies is still unknown. These challenges need to be considered while developing detection tools for WWE applications.

## 5. WHO’s Sample Collection Guidelines

According to the WHO’s guidelines [51], the specimen type can be: (a) skin lesion material, including swabs of lesion exudate, lesion roofs, and lesion crusts; (b) oropharyngeal swabs; (c) rectal and or genital swabs; (d) urine; (e) semen; (f) whole blood; (g) serum; or (h) plasma. Skin lesion material is the recommended specimen for diagnosis purposes. In addition, the oropharyngeal swab is encouraged for the laboratory confirmation of the cases. However, care is needed when drawing conclusions from results obtained using an oropharyngeal swab since limited data are available for this specimen type [51]. While serum and plasma samples are used for research purposes, they can be obtained for diagnostic applications in combination with skin lesion material. The rest of the specimen types, including rectal and genital swabs, urine, semen, and whole blood, are recommended to be collected for research purposes and are subject to ethics guidelines. The samples can be refrigerated (for 7 days) or frozen at −20 °C or below (for 60 days).

## 6. Conclusions and Prospects

The re-emergence of MPXV is a clear indication that the timely detection of viruses is instrumental in controlling the onset and spread of outbreaks. PCR is the gold standard for nucleic acid detection. Although sensitive and selective, the PCR-based MPXV detection approaches may not be feasible for resource-constrained settings. Isothermal nucleic acid amplification techniques are emerging alternatives. The development timeline of MPXV diagnostics indicates that limited progress has been made towards innovations in MPXV diagnostics, highlighting an obvious research gap. The WHO recommends the development of point-of-care (POC) devices. Internet of medical things (IoMT)-based POC devices have attracted substantial attention [80]. IoT-based MPXV detection might be a promising approach. For instance, the IoT-based detection of COVID-19 using LAMP technology has been demonstrated with satisfactory performance [81]. Similarly, another field-deployable RT-LAMP-based device for onsite virus inactivation and detection was also reported [82]. These advanced approaches can be extended to MPXV diagnostics. Although a number of nucleic acid methods based on LAMP technology have been developed, this approach requires a high temperature and six primers.

Alternatively, RPA technology can be equipped with smartphones for field applications since RPA requires two primers and the reaction can be performed at 37–42 °C. Although promising, the approach has some limitations. For instance, RPA, like PCR, can be inhibited by a high concentration of genomic DNA [83]. Furthermore, multiplex detection using RPA might be challenging, as RPA primers for different genes or targets compete for the RPA proteins. The problems can be solved by integrating RPA with microfluidic platforms where multiplex detection can be performed in separate microfluidic compartments [84,85]. The use of multiple quantum dots for different targets and coupling with DNA barcodes could be a fascinating approach to develop a POC detection system where MPXV could be distinguished from the rest of the poxviruses. Further, a separate solid-phase amplification can also overcome the problems of RPA-based multiplex detection [86]. Though RPA can be performed at a relatively low temperature, the approach still requires special temperature handling, which may limit its POC applications. To overcome this limitation, alternative strategies could be helpful, such as the use of hand warmers to control the temperature [87].

Wearable devices have found increased applications in recent years [88]. A comprehensive understanding of MPXV’s current clinical manifestations [89] and the integration of this information with smartphone apps and smartwatches might be helpful in developing screening systems for presymptomatic cases. For instance, Mishara et al. reported a comprehensive study where physiological data from smartwatches were used to predict COVID-19 presymptomatic cases [90]. Inspired by this work, many machine learning algorithms have been reported in recent studies [91,92] and are equally important for MPXV detection. It is important to know the virus’s infectiousness status after infection. The available methods solely predict the presence or absence of the virus or virus particles. A method for determining the virus’s infectiousness in infected patients or environmental samples could be a valuable addition to MPXV research. For this, immunodiagnostic methods may contribute to some extent, but they have certain limitations, especially poor selectivity for MPXV. The antigen detection methods are rapid and cost-effective but less sensitive. The same applies to MPXV immunodiagnostics. Therefore, novel MPXV antigen detection methods will be developed in the near future. Due to MPXV’s genome identity with other orthopoxviruses, finding a unique antigen is a daunting challenge. The E8L protein of MPXV is a membrane protein and is a potential target for vaccines. Recently, non-cross-reactive epitopes for MPXV were reported within the E8L protein via a computational approach [93]. It is anticipated that E8L-binding peptides could also be discovered in a similar way and could be used as a biosensing layer for the specific detection of MPXV. Further, the E8L-binding aptamers and nanobodies [94] can make valuable contributions. To the best of our knowledge, the MPXV entry receptor is still unknown; the discovery of the MPXV entry receptor and the development of MPXV sensors based on the entry receptor could be useful future developments. Novel MPXV biosensors could be developed based on photonics [95], quantum dots [96], electrochemiluminescence [97], electrochemical transduction, lab on a chip [30], CRISPR technology [98,99], and other approaches [100,101,102,103]. Introducing smart diagnostic systems based on WWE is anticipated to be a good future work to detect asymptomatic cases.

Since genome sequence data provide detailed information about the phylogenetic origin, mutations, and genomic recombination of a pathogen, in addition to PCR, it is recommended to perform the sequencing of as many samples as possible. The PCR assays developed for previous MPXV outbreaks should be reverified for the recent outbreak in order to ascertain that new mutations do not affect the target region of the PCR assays. Nucleic acid amplification tests are very sensitive and are prone to contamination; standard operating procedures (SOPs) should be strictly followed while performing these assays or developing a new technique. Most of the MPXV cases are concentrated in certain regions. The unavailability of real samples may hamper the clinical validation of the tests under development. In this regard, the transport of inactivated samples from hotspot countries should be considered to expedite the validation of the MPXV detection systems. Initially, most of the SARS-CoV-2 diagnostic tools were developed by repurposing the assays developed for previous coronavirus outbreaks. The same strategy should be considered for MPXV detection. Although MPXV is a re-emerging virus, research on MPXV detection is still not well-explored, providing room for future developments, and should be considered by the scientific community to prevent further spread of this virus.
